# Genetic and biochemical markers of hydroxyurea therapeutic response in sickle cell anemia

**DOI:** 10.1186/1471-2350-14-108

**Published:** 2013-10-09

**Authors:** Danilo Grunig Humberto Silva, Edis Belini Junior, Gisele Cristine de Souza Carrocini, Lidiane de Souza Torres, Octávio Ricci Júnior, Clarisse Lopes de Castro Lobo, Claudia Regina Bonini-Domingos, Eduardo Alves de Almeida

**Affiliations:** 1 Department of Biology, Hemoglobin and Hematologic Genetic Diseases Laboratory, Sao Paulo State University–UNESP, Sao Paulo, Brazil; 2 Department of Medicine, Sao Jose do Rio Preto Medical School–FAMERP, Sao Paulo, Brazil; 3 Hematological State Institute “Arthur de Siqueira Cavalcanti”–HEMORIO, Rio de Janeiro, Brazil; 4 Department of Chemistry and Environmental Sciences, Sao Paulo State University–UNESP, Sao Paulo, Brazil

**Keywords:** Hemoglobin S, Beta-S-gene cluster haplotypes, Oxidative stress, Antioxidant capacity

## Abstract

**Background:**

Sickle cell anemia (SCA) presents a complex pathophysiology which can be affected by a number of modifying factors, including genetic and biochemical ones. In Brazil, there have been no studies verifying β^S^-haplotypes effect on oxidative stress parameters. This study evaluated β^S^-haplotypes and Hb F levels effects on oxidative stress markers and their relationship with hydroxyurea (HU) treatment in SCA patients.

**Methods:**

The studied group was composed by 28 SCA patients. Thirteen of these patients were treated with HU and 15 of them were not. We used molecular methodology (PCR-RFLP) for hemoglobin S genotype confirmation and haplotypes identification. Biochemical parameters were measured using spectrophotometric methods (Thiobarbituric-acid-reactive substances and Trolox equivalent antioxidant capacity levels, catalase and GST activities) and plasma glutathione levels by High-performance liquid chromatography coupled to electrochemical detection.

**Results:**

We found the highest frequency of Bantu haplotype (48.2%) which was followed by Benin (32.1%). We observed also the presence of Cameroon haplotype, rare in Brazilian population and 19.7% of atypical haplotypes. The protective Hb F effect was confirmed in SCA patients because these patients showed an increase in Hb F levels that resulted in a 41.3% decrease on the lipid peroxidation levels (r =−0.74, p=0.01). Other biochemical parameters have not shown differential expression according to patient’s haplotypes. Bantu haplotype presence was related to the highest lipid peroxidation levels in patients (p < 0,01), but it also conferred a differential response to HU treatment, raising Hb F levels in 52.6% (p = 0.03) when compared with the group with the same molecular profile without HU usage.

**Conclusions:**

SCA patients with Bantu haplotype showed the worst oxidative status. However these patients also demonstrated a better response to the treatment with HU. Such treatment seems to have presented a “haplotype-dependent” pharmacological effect.

## Background

Sickle cell anemia (SCA) is a chronic and progressively debilitating medical condition featuring ongoing hemolytic anemia and recurrent acute vaso-occlusive events [1]. It is characterized by a clinical course highly variable, ranging from death in early childhood [2] to a normal life span with few complications [3]. This feature reflects the complex pathophysiology of SCA which can be affected by a number of modifying factors including haplotype of β-globin gene cluster [4], coinheritance of polymorphisms associated with clinical aspects [5,6] and treatment response [7], hemoglobin fetal (Hb F) levels [8], chronic inflammation and oxidative states [9,10] as well as gender [4].

There are five distinct haplotypes linked to the β^S^-mutation and they are known as Benin (Ben), Bantu or Central African Republic, Senegal (Sen), Cameroon (Camer) and Indian-Arab haplotypes. These ones are classified according to the geographical region in which they were originally identified [11,12]. Analysis of β^S^ polymorphisms is of genetic and anthropologic interest, but it may also be related to disease severity as well as variations in drug response [13,14]. Bantu haplotype has been associated with more severe disease outcome and a high organ damage incidence. Benin haplotype has been associated with intermediate disease severity. On the other hand, Senegal and Indian-Arab haplotypes have been associated with milder disease severity [13,15] due to their higher Hb F levels related to the C → T mutation at position -158 *Xmn*I in the ^G^γ-globin gene promoter region [15].

Hydroxyurea (HU) administration seems to be the best available treatment option for SCA patients [1,16,17]. HU is an antineoplastic drug which its main pharmacological action is to increase Hb F levels. It has other potentially beneficial effects including improved nitric oxide (NO) metabolism, reduced red cell–endothelial interaction and decreased erythrocyte density [1]. Although highly effective for most SCA patients, there is a considerable inter-patient variability creating a broad spectrum of Hb F induction [1,18]. HU mechanisms of action for Hb F induction remain incompletely understood. Hb F induction by HU has been correlated to cell cycle inhibition leading to activation of stress erythropoiesis [1,19-21]. Other studies have suggested that Hb F induction by HU is mediated more specifically via nitric oxide-dependent transcriptional mechanisms [22,23] and cyclic nucleotides [24,25] and initial evidence for some epigenetic regulation [26].

Many studies have been carried out trying to establish a relation between β^S^-haplotypes and SCA phenotype. These haplotype-phenotype associations are not definitely established and no clear correlation has emerged [6,27-29] to date, though. In Brazil, there have been no studies verifying β^S^-haplotypes effect on oxidative stress parameters. Therefore this work aimed at studying β^S^-haplotype effects and Hb F levels on oxidative stress markers and their relationship with HU treatment.

## Methods

### Patients

Eligible patients were 10 years or older at the beginning of the study and they were diagnosed with SCA. They all had access to the same medication protocol. The studied group was composed by 28 SCA patients (11 males and 17 females; mean age: 27.7 years old; range: 10-65 years old) in clinical follow-up in Sao Jose do Rio Preto (SP) and Rio de Janeiro (RJ). All the patients are from the southeast region of Brazil.

All SCA patients were screened using a questionnaire. Pregnant, smokers or drinkers were excluded from the study, as well as anyone who had had a stroke, pain and/or hemolytic crisis or had received blood transfusion within two months prior to the start of the study. The medications used by SCA patients were previously checked and the ones taking any other medication known to affect the parameters analyzed (such as acetylsalicylic acid, antibiotics or vitamins) within 24 h of sample collection were also excluded. All subjects gave their informed consent and the study was reviewed and ethically approved by the Data Safety Monitoring Board (DSMB) according to Brazilian Regulations and Ethical Committee of Sao Paulo State University (0015.0.229.000-09).

### Biological samples

Blood samples (11 mL) were collected through venipuncture in heparinized and ethylenediamine tetraacetic acid (EDTA) tubes. The heparinized blood (7 mL) was incubated for 20 min at 37°C and then centrifuged at 206 g for 20 min to separate plasma for Thiobarbituric-acid-reactive substances (TBARS) and Trolox equivalent antioxidant capacity (TEAC) analysis. The EDTA sample fraction (4 mL) was aliquoted: 2 mL used for the hemoglobinopathies tests, genotypic determination and catalase (CAT) and glutathione *S*-transferase (GST) enzymatic activities analysis and the other 2 mL were submitted to centrifugation at 825 g for 10 min to obtain plasma and then were frozen at−80°C for glutathione (GSH) levels determination.

### Hemoglobin phenotypes, genotypes and β^S^-globin haplotypes

Hb identification was performed using electrophoresis on cellulose acetate pH 8.4 and agar electrophoresis at pH 6.2. Hb fraction quantification was obtained using high performance liquid chromatography (HPLC) by the automated VARIANT™ equipment (Bio-Rad Laboratories, CA, USA) [30]. Cell morphology microscopic analysis was performed on the stained blood using May-Grünwald-Giemsa. In all patient samples, Hb S genotype was developed by molecular analysis using PCR-RFLP. The segment amplification that encodes β^S^ gene was accomplished by specific primers and amplicon was cleaved by the *Dde*I restriction endonuclease (New England BioLabs, MA, USA) [31]. Beta globin haplotypes were determined through the PCR-RFLP analysis of the following polymorphic restriction sites: γG (Hind III), γA (Hind III), ψβ (Hinc II), 3′ψβ (Hinc II) and 5′β (Hinf I), as previously described [32].

### Biochemical analysis

Lipid peroxidation levels were assessed in heparinized plasma using TBARS assay [33]. Antioxidant capacity was also determined in heparinized plasma samples according to their equivalence to Trolox (6-hydroxy-2,5,7,8-tetramethychroman-2-carboxylic acid) [34]. For total GST activity, blood samples were diluted in a 3.5 μM 2-mercaptoethanol 10 μM NADP 2.7 mM EDTA hemolyzing solution (1:20, v/v) and then assayed using 1-chloro-2,4-dinitrobenzene (CDNB) as substrate at 340 nm. The assay was carried out in 0.2 M K-phosphate buffer pH 6.5, 1 mM CDNB, 1 mM GSH (ϵ = 9.6 mM^-1^ cm^-1^) [35]. For CAT activity analysis, blood samples were diluted in ultrapure water (1:50, v/v) and then 10 μL were used to measure CAT activity, by the decrease in absorbance at 240 nm (ϵ = 0.04 mM^-1^ cm^-1^) due to consumption of H_2_O_2_ (10 mM H_2_O_2_ in 1 M Tris–HCl buffer pH 8.0 containing 5 mM EDTA) [36].

GSH concentration was determined in EDTA plasma samples using HPLC coupled to a coulometric electrochemical detector (Coulochem III ESA, Bedford, MA) [37]. Under these conditions, GSH clearly eluted in ~ 6 min. GSH was extracted from the plasma samples by adding perchloric acid to the plasma sample (10% final concentration). After vigorous stirring and remaining 10 min on ice, the mixture was centrifuged at 825 g for 10 min at 4°C. The extract was then filtered through Millex syringe filter units (0.22 μm) and directly injected into the HPLC system. The calculations were based on a calibration curve previously constructed by injecting authentic GSH standards into HPLC system.

### Statistical analysis

Statistical analysis was performed in groups with at least three individuals using the Statistica 9.0 software (Statsoft Inc.). Data were tested regarding normality and homogeneity of variances assumptions according to Shapiro-Wilk test and Levene’s test, respectively. Groups that met the assumptions (parametric data) were compared by applying t test or one-way ANOVA followed by Fisher’s post hoc. Those groups that did not meet the assumptions (non-parametric data) were compared by Mann–Whitney test or Kruskal-Wallis followed by Dunn’s post hoc test. In order to assess association degree between the studied variables, we used Pearson’s correlation for parametric data and Spearman’s rank correlation for non-parametric data. In order to assess age and gender influence on the values of oxidative stress markers, we classified SCA patients into two age groups (≤ 20 and > 20 years) and we applied factorial ANOVA.

Data were expressed as mean ± standard deviation and p < 0.05 was considered statistically significant.

## Results

Through β^S^-haplotypes molecular analysis we found nine different combinations of restriction sites, resulting in the following specific combinations: Bantu, Benin, Cameroon and three atypical. The atypical patterns were classified by the numbers 1, 2 and 3, they do not fall into any of the classifications previously reported in the literature (Table 1).

**Table 1:** **Characterization of atypical β**^**S**^**-haplotypes alleles**

**Restriction sites**						
**β**^**S**^**-Haplotypes**	***Xmn *I**	***Hind *III**		***Hinc *II**		***Hinf *I**
	5′γ ^G^	γ ^G^	γ ^A^	Ψβ	3′ψβ	5′ β
Atypical 1	-	-	-	-	-	-
Atypical 2	-	+	-	-	+	-
Atypical 3	-	-	+	-	+	-

We identified eight (28.5%) patients with haplotype Bantu/Bantu, 10 (35.7%) Bantu/Benin, two (7.1%) Benin/Benin, one (3.6%) Benin/Cameroon, one (3.6%) Bantu/Atypical 1, one (3.6%) Benin/Atypical 1, one (3.6%) Benin/Atypical 2, one (3.6%) Benin/Atypical 3 and three (10.8% ) Atypical 2/Atypical 2. From 56 chromosomes analyzed, the allelic frequency observed was: 27 (48.2%) alleles Bantu, 18 (32.1%) Benin, one (1.8%) Cameroon and 10 (19.7%) Atypical, from the atypical ones, two (3.6%) Atypical 1, seven (12.5%) Atypical 2 and one (1.8%) Atypical 3.

For biochemical parameters assessment, firstly we checked whether age and gender could influence the values of studied markers (TBARS and TEAC levels, GST and CAT enzyme activities and plasma GSH levels) to avoid biases. We found no statistically significant difference for any of the evaluated parameters, as shown in Table 2.

**Table 2:** Analysis of the age and gender interference on the biochemical markers values in SCA patients

	**Age**^**#**^		P values*	**Gender**^**#**^		P values*
	**≤ 20 years n = 09**	**> 20 years n = 19**		**Male n = 11**	**Female n = 17**	
**TBARS (ng/mL)**	1452.94 ± 699.00	1577.07 ± 539.85	0.4950	1345.94 ± 413.13	1660.90 ± 655.72	0.0719
**TEAC (mM)**	1.97 ± 0.21	2.03 ± 0.15	0.6737	2.01 ± 0.23	2.01 ± 0.12	0.7292
**GST (U/mL)**	1.77 ± 0.94	1.51 ± 0.49	0.2394	1.50 ± 0.56	1.64 ± 0.72	0.3757
**CAT (U/mL)**	1660.80 ± 525.41	1912.16 ± 517.83	0.0957	1831.30 ± 634.04	1831.40 ± 461.03	0.7524
**GSH (μM)**	0.74 ± 0.49	0.70 ± 0.39	0.3644	0.59 ± 0.52	0.79 ± 0.31	0.2791

*Comparisons were made by factorial ANOVA.

^#^There were no significant interactions between independent variables: age and gender (p > 0.05).

The influence of haplotypes and HU treatment over Hb F concentration and on biochemical markers was determined by subgroups formation - haplotype and HU use (+ HU) and haplotype without HU use (–HU). The values and/or mean of ana\lyzed parameters according to subgroup are presented in Table 3.

**Table 3:** **Descriptive analysis of the β**^**S**^**-haplotypes interference in the phenotypic expression of SCA patients**

		**Parameters**					
		**Hb F (%)**	**TBARS (ng/mL)**	**TEAC (mM)**	**GST (U/mL)**	**CAT (U/mL)**	**GSH (μM)**
**Haplotypes (+HU)**	**n**						
Bantu/Bantu	2	1.95	1201.18	2.17	1.26	1742.95	1.30
Bantu/Benin	6*	17.42	1066.26	2.03	1.50	2294.01	0.62
Benin/Benin	1	5.2	1616.62	2.17	1.44	2278.17	1.10
Benin/Camer	0	-----	-----	-----	-----	-----	-----
Bantu/Atp1	0	-----	-----	-----	-----	-----	-----
Benin/Atp1	1	1.8	1524.30	2.20	1.20	1531.69	0.19
Benin/Atp2	1	8.4	1012.00	2.01	2.00	1084.51	0.22
Benin/Atp3	1	7.3	1216.92	2.10	1.92	1880.28	0.88
Atp2/Atp2	1	11	1308.00	1.97	1.51	1866.20	0.37
**Haplotypes (−HU)**							
Bantu/Bantu	6*	6.78	2284.33	1.93	2.03	1656.10	0.77
Bantu/Benin	4*	6.68	1815.50	2.09	1.27	1842.43	0.84
Benin/Benin	1	2.1	1222.00	2.09	1.65	2570.42	0.74
Benin/Camer	1	4.8	934.00	1.90	2.00	996.48	0.70
Bantu/Atp1	1	3.2	1287.00	1.95	1.18	2017.61	0.87
Benin/Atp1	0	-----	-----	-----	-----	-----	-----
Benin/Atp2	0	-----	-----	-----	-----	-----	-----
Benin/Atp3	0	-----	-----	-----	-----	-----	-----
Atp2/Atp2	2	7.45	1576.00	1.77	1.45	1248.24	0.32

(+HU): patients treated with HU; (−HU): patients not treated with HU; Camer: Cameroon; Atp: atypical.

*Subgroups subject to statistical comparisons.

Between the subgroups submitted to statistical comparisons, we assessed haplotypes effect on SCA phenotypic expression markers, comparing Bantu/Bantu (–HU) with Bantu/Benin (–HU) and we observed no statistical difference (Table 4). In order to prove the contribution of HU use on these markers, according to haplotypes subgroups, we compared Bantu/Benin (+HU) with Bantu/Benin (–HU) and found an increase in Hb F levels in the treated subgroup (p < 0.01) and consequent lipid peroxidation reduction (p = 0.03) (Table 5).

**Table 4:** Influence of Bantu and Benin haplotypes on SCA phenotypic expression

**Modulators**	**Bantu/Bantu (−HU)**	**Bantu/Benin (−HU)**	P values*
	**n = 06**	**n = 04**	
**Hb F (%)**	6.78 ± 3.60	6.67 ± 6.37	0.9731
**TBARS (ng/mL)**	2284.33 ± 435.50	1815.50 ± 334.80	0.1074
**TEAC (mM)**	1.92 ± 0.13	2.08 ± 0.18	0.1465
**GST (U/mL)**	2.03 ± 1.02	1.27 ± 0.63	0.2245
**CAT (U/mL)**	1656.10 ± 413.96	1842.42 ± 397.81	0.4993
**GSH (μM)**	0.77 ± 0.37	0.84 ± 0.62	0.8317

(−HU) patients not treated with HU. Mean ± standard deviation.

* Comparisons were made by Mann–Whitney test.

**Table 5:** Influence of the HU use in SCA patients with the Bantu/Benin haplotype

**Modulators**	**Bantu/Benin (+HU)**	**Bantu/Benin (−HU)**	P values*
	**n = 06**	**n = 04**	
**Hb F (%)**	17.41 ± 3.10	6.67 ± 4.37	0.0069^**#**^
**TBARS (ng/mL)**	1066.26 ± 495.09	1815.50 ± 334.80	0.0303^**#**^
**TEAC (mM)**	2.02 ± 0.16	2.08 ± 0.18	0.6124
**GST (U/mL)**	1.49 ± 0.62	1.27 ± 0.63	0.5925
**CAT (U/mL)**	2294.01 ± 297.29	1842.42 ± 397.81	0.0725
**GSH (μM)**	0.62 ± 0.37	0.84 ± 0.62	0.4981

(−HU) patients not treated with HU. (+HU) patients treated with HU.

*Comparisons were made by Mann–Whitney test.

^**#**^Indicates statistical difference (p < 0.05).

**Figure 1:**
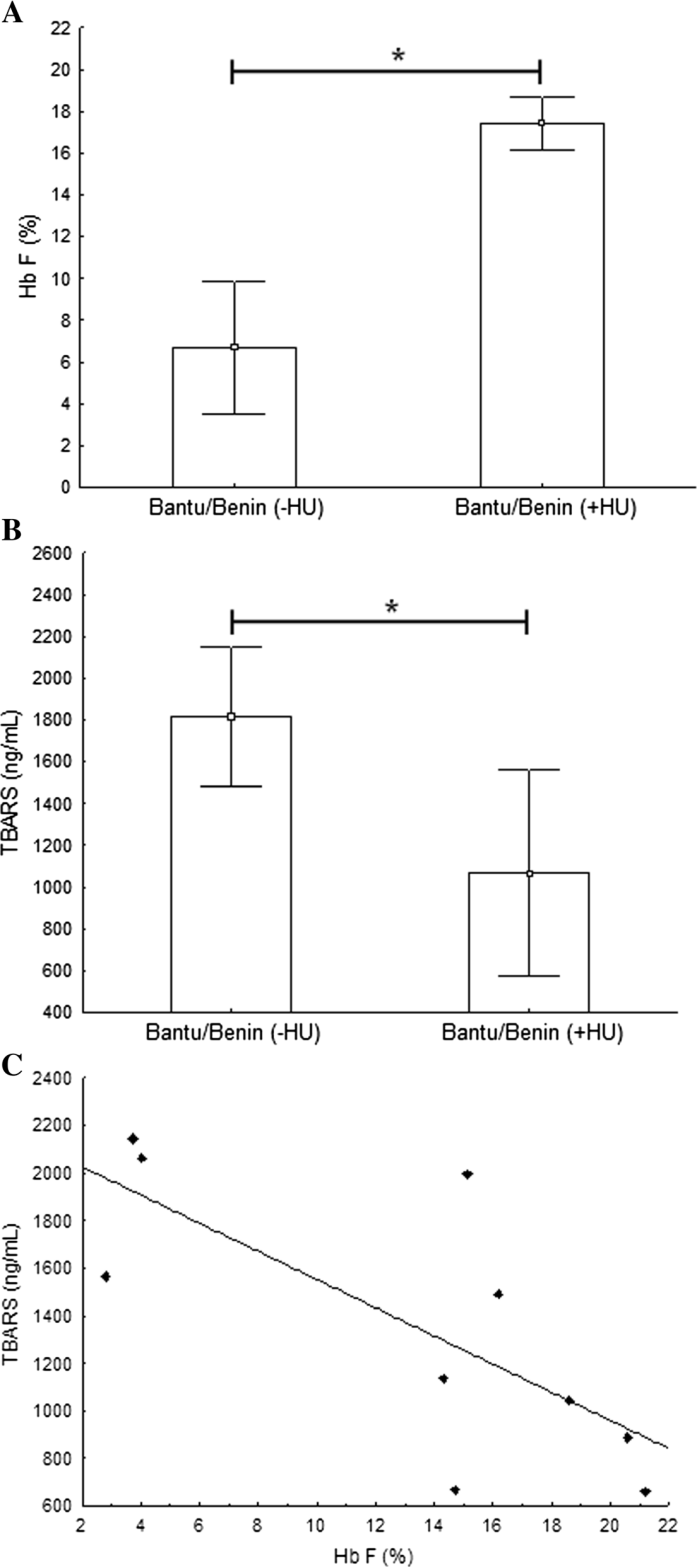
**Hb F and lipid peroxidation levels in SCA patients with Bantu/Benin haplotype. A)** Hb F levels were about 2.6 times higher in patients under HU treatment compared to those not treated (p = 0.0069; Mann–Whitney test). **B)** Lipid peroxidation levels showed 1.7 times lower in those patients on HU usage (p = 0.0303; Mann–Whitney test). **C)** Negative linear correlation between Hb F and lipid peroxidation levels (r = −0.74; p = 0.0156; Spearman’s rank test).

The association degree among the studied markers showed that in patients with the same β^S^-haplotype (Bantu/Benin), HU promoted an increase of 61.7% in Hb F values (Figure 1A) and a decrease of 41.3% in lipid peroxidation levels (Figure 1B), according to a negative correlation found between these markers (r =−0.74, p = 0.01) (Figure 1C). The other evaluated biochemical parameters showed no differential expression or association.

Bantu haplotype is associated with the worst clinical outcomes in SCA. Therefore, to better address Bantu haplotype influence on oxidative stress markers and HU usage, we classified the patients into four sample groups:

Group I. Patients with Bantu haplotype at least one chromosome without HU treatment. The haplotypes that comprised this group were Bantu/Bantu, Bantu/Benin and Bantu/Atp1;

Group II. Patients with Bantu haplotype at least one chromosome and under HU treatment. The haplotypes were Bantu/Bantu and Bantu/Benin;

Group III. Patients without the Bantu haplotype a HU usage. This group was composed by haplotypes Benin/Benin, Benin/Camer, Atp2/Atp2;

Group IV. Patients without Bantu haplotype in any chromosome, but under HU usage. The haplotypes were Benin/Benin, Benin/Camer, Atp2/Atp2.

Table 6 summarizes obtained results from the comparisons between such groups for all evaluated parameters.

**Table 6:** Relationship between the Bantu haplotype and HU treatment on SCA patients

	**Sample groups**				P values*
	**Group I**	**Group II**	**Group III**	**Gruop IV**	
	**n = 11**	**n = 08**	**n = 04**	**n = 05**	
**Hb F (%)**	6.42 ± 4.45^*a*^	13.55 ± 7.66^*b*^	5.45 ± 5.42^*a*^	6.74 ± 3.46^*a*^	0.0388
**TBARS (ng/mL)**	2023.18 ± 490.74^*a*^	1099.99 ± 455.70^*b*^	1327.00 ± 463.9^*b*^	1335.57 ± 241.94^*b*^	0.0009
**TEAC (mM)**	1.99 ± 0.16	2.06 ± 0.15	1.88 ± 0.27	2.09 ± 0.10	0.2296
**GST (U/mL)**	1.68 ± 0.90	1.44 ± 0.55	1.64 ± 0.53	1.61 ± 0.34	0.8974
**CAT (U/mL)**	1756.72 ± 385.98	2156.25 ± 540.32	1515.85 ± 759.98	1728.17 ± 446.54	0.1758
**GSH (μM)**	0.81 ± 0.44	0.79 ± 0.45	0.52 ± 0.24	0.55 ± 0.41	0.4987

*Different letters indicate statistical differences (ANOVA followed by Fisher’s post hoc test).

Group I: Bantu (−HU), Group II: Bantu (+ HU), Group III: any haplotype except Bantu (−HU), Group IV: any haplotype except Bantu (+ HU).

**Figure 2:**
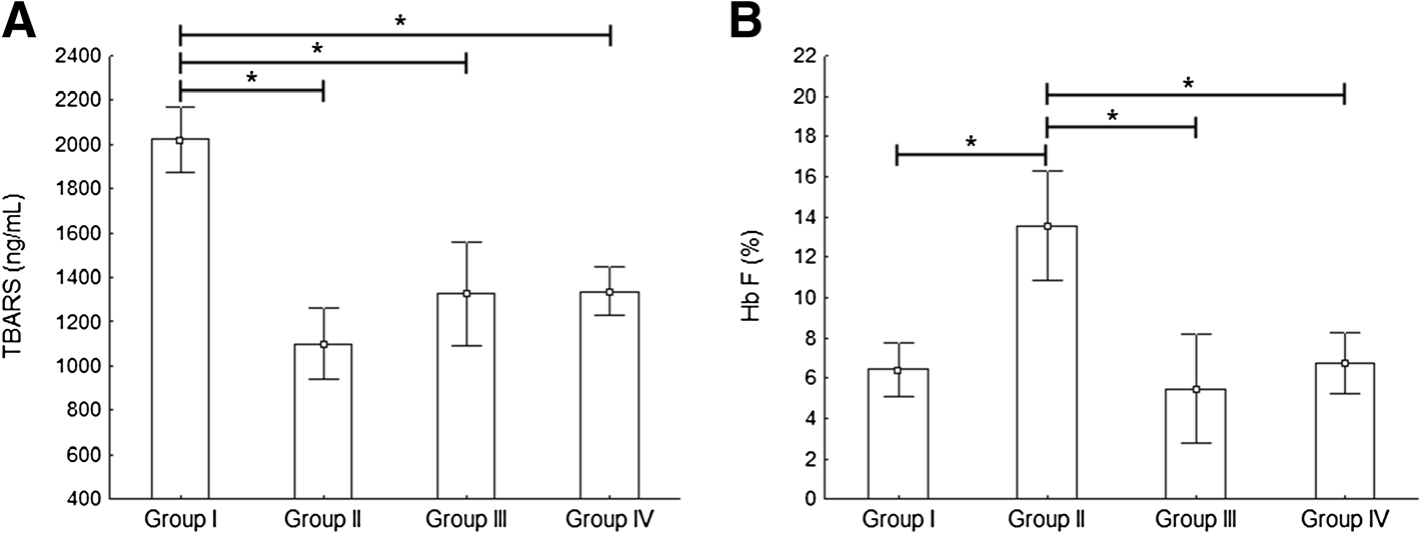
**Analysis of Bantu haplotype effect and HU use on SCA phenotypic expression modulators. A)** Lipid peroxidation showed its highest mean value in Group I compared to the others groups. **B)** Higher Hb F levels in patients of Group II compared to group with the same molecular profile not treated (Group I) and to the others evaluated groups. *Indicates statistical difference (ANOVA followed by Fisher’s post hoc test). Group I: Bantu (−HU), Group II: Bantu (+ HU), Group III: any haplotype except Bantu (−HU), Group IV: any haplotype except Bantu (+ HU).

The haplotype sample group analysis also showed significant differences only in the Hb F and lipid peroxidation markers. Bantu haplotype presence was related to the highest lipid peroxidation levels in patients (p < 0.01) (Figure 2A), but also, it conferred a differential response to HU treatment, raising Hb F levels in 52.6% (p = 0.03) when compared with the group with same molecular profile not treated (Group I). This treatment response was not observed in patients without Bantu haplotype (Figure 2B).

## Discussion

Although SCA is one of the first disorders to be clearly defined at molecular level, genetic understanding of the basis for disease expression variability is still unclear [38]. Since β^S^-haplotypes discovery as genetic modulators of phenotypic expression in SCA, several studies have been developed to determine haplotypes effect on SCA patients hematological and clinical features [13,27,38-41], but studies associating haplotypes with oxidative stress markers are scarce. This study, to our knowledge, yields a unique opportunity in which both genetic factor (β^S^-haplotypes) and oxidative stress markers were simultaneously measured and correlated with Hb F levels and HU use in Brazilian SCA patients.

We found a higher frequency of Bantu haplotype followed by Benin. This distribution of β^S^-haplotypes was similar to other studies with Brazilian SCA patients from southeast region [39-44]. The chromosomes majority with β^S^ gene has one of the five common haplotypes, although in every large series of SCA patients there is a minority of chromosomes (5 ± 10%) usually referred as “atypical” haplotypes [45]. We found 19.7% of atypical haplotypes, higher frequency than it is expected. None of the identified haplotypes during the study have had presence of *Xmn*I polymorphic site, neither those haplotypes already described in the literature nor the atypical ones. Therefore, other genetic polymorphisms not targeted in this study should be involved in high Hb F levels obtained in SCA patients not treated with HU.

Bantu/Benin haplotype was the most frequent. Therefore in these patients, we confirmed Hb F protective effect provided by HU use. Once increasing Hb F levels resulted in a decrease of the lipid peroxidation levels in accordance with our recent publications [44,46]. The protective effect is due to the increase in Hb F concentration that either inhibits or retards Hb S polymerisation [47], leading to a decreased intravascular sickling and an increasing nitric oxide bioavailability [48]. These alterations result in a decreased oxidative stress with markedly decreased lipid peroxidation and increased activity⁄levels of antioxidants (SOD, GPx, catalase, and GSH) [48]. This antioxidant response was not observed though, according to the haplotype profile.

Bantu haplotype presence was related to the highest lipid peroxidation levels in patients, corroborating with the results obtained by Rusanova et al. [49]. The authors showed that SCA patients with Senegal and Indian-Arab alleles had the mild clinical outcomes associated with low oxidative status, whereas high oxidative stress was related to Benin and Bantu haplotypes, consequent severe phenotypes. On the other evaluated parameters (TEAC levels, CAT and GST activities and plasma GSH levels), we have not observed any significant haplotype influence. Thus, oxidative stress biomarkers analysis may be important in clinical condition evaluation of SCA patients, furthermore in therapeutic response monitoring among SCA patients under HU use.

Currently, many researches aimed at identifying inter-individual genetic variations, underlying different pharmacological responses to drug use [50]. In SCA, this paradigm is being applied to elucidate vascular complications pathogenesis and to develop individualized therapies [6]. However, there is no stated relationship in the literature between differential response to HU treatment according to β^S^-haplotypes in SCA patients. Vicari et al. [51] showed, in contrast to previous reports [52-54], a significant increase in Hb F levels in SCA patients with Bantu haplotype after HU use, similar HU pharmacological response that we obtained in our studied group. As it is estimated that 40% of the patients do not respond to HU treatment [55] and Bantu haplotype is the most frequent in Brazilian SCA patients, this HU differential response should be carefully interpreted, according to Vicari et al. [51].

We hypothesized that this “haplotype-dependent” pharmacological effect of HU is due to the “highest stress erythropoiesis stimulation” in SCA patients with Bantu haplotype. The presence of Bantu haplotype is associated with a hyperoxidative status and consequent higher hemolytic levels and lower Hb concentrations, characteristics known to increase the circulating erythropoietin concentrations, which in turn stimulates erythropoiesis [56-58]. Based on HU cytotoxic effect which is beneficial in many ways; it targets rapidly dividing cells, which in red cells tend to be those ones with a high Hb S levels and favors the production of red cells with a high Hb F levels, as these levels tend to arise from red cells that divide less rapidly [59]. This way, SCA patients with Bantu haplotype under HU use would have higher erythropoiesis stimulation, favoring production of red cells with a high Hb F levels. This hypothesis agrees with the observations from Gordeuk et al. [60]. The authors confirmed by multiple linear regression that lower hemoglobin concentration was correlated with higher erythropoietin concentration and higher Hb F percentage among sickle cell disease patients. Therefore, even with a small sample size, our results have left perspectives for further studies to better address this hypothesis.

## Conclusion

We provided evidence that Bantu haplotype presence seems to be an important predictor factor of oxidative stress and of differential response to HU use in SCA patients. We confirmed a hyperoxidative status among SCA patients. This status should be considered, at least partially, on clinical manifestations variety of these patients. Thus, the use of oxidative stress biomarkers may be important in the evaluation of clinical condition of SCA patients, furthermore in therapeutic response monitoring among SCA patients under HU use. We also suggest that the development of therapies to improve the redox status would be beneficial to reduce the severity of SCA.

## Competing interests

The authors declare no competing financial or other relationship with other people or organizations interests.

## Authors’ contributions

DGHS: data design, data acquisition, data analysis, statistical analysis, data interpretation and manuscript preparation. EBJ: technical assistance on molecular, biochemical and statistical analysis. GCSC: technical assistance in the standardization of molecular biology analysis. LST: technical assistance on biochemical analysis. ORJ: data provision and critical review of manuscript. CLCL: data provision and critical review of manuscript. CRBD: study concept and design and critical review of manuscript. EAA: study concept and design, guidance on standardization of the biochemical methods and critical review of the manuscript. All authors read and approved the final manuscript.

## Pre-publication history

The pre-publication history for this paper can be accessed here:

http://www.biomedcentral.com/1471-2350/14/108/prepub
